# Classification of Two Class Motor Imagery Tasks Using Hybrid GA-PSO Based *K*-Means Clustering

**DOI:** 10.1155/2015/945729

**Published:** 2015-04-20

**Authors:** Suraj, Purnendu Tiwari, Subhojit Ghosh, Rakesh Kumar Sinha

**Affiliations:** ^1^Electrical and Electronics Engineering, Birla Institute of Technology, Mesra, Ranchi 835215, India; ^2^M. Tech., Computer Technology, National Institute of Technology, Raipur 492001, India; ^3^Electrical and Electronics Engineering, National Institute of Technology, Raipur 492001, India; ^4^Birla Institute of Technology, Mesra, Ranchi 835215, India

## Abstract

Transferring the brain computer interface (BCI) from laboratory condition to meet the real world application needs BCI to be applied asynchronously without any time constraint. High level of dynamism in the electroencephalogram (EEG) signal reasons us to look toward evolutionary algorithm (EA). Motivated by these two facts, in this work a hybrid GA-PSO based *K*-means clustering technique has been used to distinguish two class motor imagery (MI) tasks. The proposed hybrid GA-PSO based *K*-means clustering is found to outperform genetic algorithm (GA) and particle swarm optimization (PSO) based *K*-means clustering techniques in terms of both accuracy and execution time. The lesser execution time of hybrid GA-PSO technique makes it suitable for real time BCI application. Time frequency representation (TFR) techniques have been used to extract the feature of the signal under investigation. TFRs based features are extracted and relying on the concept of event related synchronization (ERD) and desynchronization (ERD) feature vector is formed.

## 1. Introduction

A brain-computer interface (BCI) is a system that translates human thoughts into an action via a control signal. Electroencephalogram (EEG) is the most widely used biological signal used for operating a BCI system since it has relatively short time constants and offers relatively high temporal resolution among noninvasive methods [1–3]. Numerous works over the past few decades have revealed that EEG signal recorded from the scalp are basis for BCIs [4]. Recently, development of brain-controlled devices have received a great attention because of their ability to bring mobility back to people with devastating neuromuscular disorder and improve the quality of their life [5]. Rapid increase in the volume and pace of BCI research is a result of concern and effort of the BCI groups to provide better opportunity to people who are in “locked in state” because of neuromuscular disorders. This has further increased due to the advancement in technology and allied fields of BCI [6, 7]. A BCI system is considered the most advanced neurofeedback system available [8–10]. Transferring the BCI from laboratory condition to real world application needs BCI to be applied asynchronously without any time constraint [11]. To achieve the same, ongoing brain activities are analyzed continuously irrespective of state, that is, control or noncontrol. An asynchronous BCI system is independent of the cue based manner [12].

Asynchronous scheme of BCI system being independent of cue depends on clustering for classification problem. Clustering techniques have been extensively used for classifying EEG signals [13]. Initial approach to develop asynchronous system was presented in late 1990s [8, 14]. High level of dynamism in the EEG signal reasons us to look toward the technique, which could address complexities such as dynamism and uncertainty. Evolutionary algorithms (EA), which are known to solve complex engineering problems, have been widely applied in BCI research in different aspects such as feature selection [15–17] and dimension reduction [16, 18]. Genetic algorithm (GA), particle swarm optimization (PSO), ant colony optimization (ACO) are EA methods which have attracted wide attention because of self-learning and population based search capabilities [16]. In this work, we have used GA, PSO, and hybrid GA-PSO based *K*-means clustering to distinguish two class motor imagery (MI) tasks. These EA based techniques have been used to select the initial cluster centres for *K*-means clustering. The hemispheric asymmetry reflected for the MI based task is exploited to differentiate the specific tasks using *K*-means clustering based on GA, PSO, and hybrid GA-PSO optimization techniques. Time-frequency representation (TFR) based features are extracted and relying on the concept of event related synchronization (ERS) and event related desynchronization (ERD) [19–24] feature vector is formed. We have used asynchronous approach of BCI to classify MI based two class tasks recorded in synchronous approach of BCI. The feature vector extracted using TFR constitutes the data population, which is classified using hybrid GA-PSO based *K*-means clustering along with GA and PSO based *K*-means clustering. To the best of the knowledge of the author's, hybrid GA-PSO based technique has not been used in classification of two classes MI tasks.

The next section deals with the methodology used with emphasis on experimental setup and various signal processing techniques used in this experiment. Paradigm of the experiment and extraction of spectral information from the recorded EEG signal are explained briefly.  Section 3 presents the results of different optimization based *K*-means in terms of accuracy and speed of convergence in addition to comparison with the conventional *K*-means clustering. Finally conclusion and discussions are presented in Section 4.

## 2. Materials and Methods

**Figure 1:**
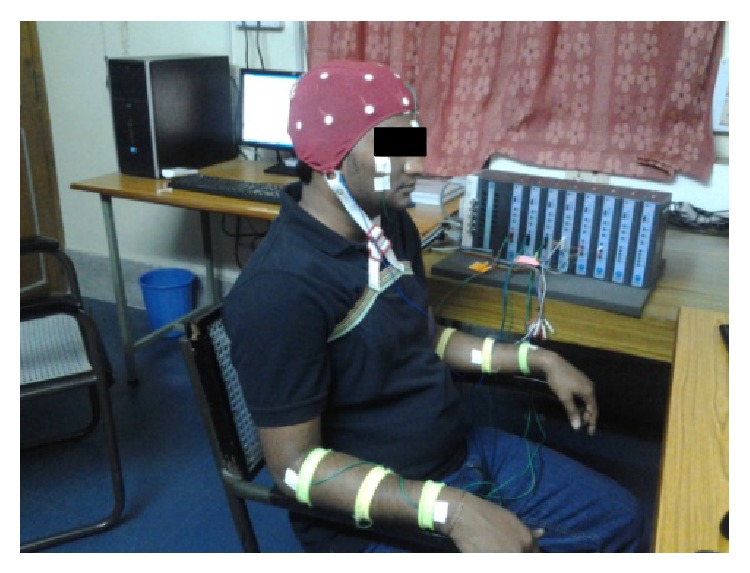
Recording setup for the BCI experiment.

Nine right-handed subjects (20–28 years, all males) are involved in this experiment. All the subjects were novice to the BCI systems and had no previous experience with any biomedical signal recording. Before the experiment, it is confirmed that all subjects are physically and mentally fit and had no prior medical history of any neurological disorder. All the subjects are given briefing of the work and tasks that they are supposed to perform. Subjects are informed that experiment involves noninvasive method and it has no adverse effect on the subjects in any form. Prior written consent is taken for the recording adhering to the ethical guidelines for biomedical research involving human research mentioned in [25].  Figure 1 displays the recording setup for the undertaken experiment.

A paradigm is prepared for two types of motor imagination tasks along with rest state. The duration of single paradigm operation (trial) was twelve seconds in which the subjects are asked to stay in the relaxed state for the first six seconds. At the sixth second alerting beep sound is used. After one second of the beep sound, a cue for 1.25 seconds is set during which subjects are instructed to imagine a movement of the left- or right-hands in accordance with the arrow direction, which is set to appear on the screen kept at a distance of about two feet from the subject. Subjects are instructed to apply just enough effort/force so that they could experience an imagination of moving left- or right-hands without any real movement. Duration of cue is chosen as 1.25 seconds in accordance with the protocol mentioned in [15, 26, 27]. Particular cue appeared randomly in order to eliminate any chance of predicting the preceding cue. For every subject two sessions of recordings are planned. Every session is planned for eight runs. Each run comprised twenty trials with different task, that is, imagination of left and right arms along with state of rest. Thus each run consists of twenty left and twenty right imagination trials.

### 2.1. Signal Recording

In the experiment, six channels bipolar recording are done, that is, three for motor cortex area (using three bipolar channels C3, C4, and Cz) and rest three channels are used to detect unwanted movement and to eliminate portions of recordings so as to reduce the influence of physiological noise. Electrooculogram (EOG) is recorded bipolarly in referential manner using three electrodes, one placed medially above and two placed laterally below the left and right eyes, respectively. In addition to this EMG is recorded from the *m* extensor digitorum communis of the right and left arms as mentioned in [27], to detect unwanted movements, if there any during recording. For recording EMG and EOG gold plated electrodes are used and EEG signal is recorded using EEG cap with 21 Ag electrodes. All signals, including three channel EEG signals, EOG, and two channels of EMG, are sampled at 500 Hz. To filter out mains hum from the 50 Hz power line a notch filter is used. Signals recorded from all six channels are grouped into three categories as right imagery, left imagery, and rest state producing eighteen sets of one-dimensional data.

### 2.2. Signal Processing

These data are further grouped as per trial and run producing twelve sets of three-dimensional data of size 625 × 10 × 8 corresponding to left and right imagery tasks for each channel and six sets of three-dimensional data of size 3000 × 20 × 8 corresponding to rest state for each channel. This size is as per trial duration of 1.25 seconds for imagery tasks with sampling frequency of 500 Hz, thus resulting in 625 data points in one trial, which is also chosen as epoch length. In every session, there are eight runs, each consisting of ten right and ten left imagery tasks. For the rest state, duration of six seconds with sampling frequency of 500 Hz agrees with the size for the data corresponding to rest state. Considering every rest state as a task, twenty sets of rest task are obtained for each session. Eighteen sets of three-dimensional data (*l* × *m* × *n*) where *l*, *m*, and *n* represent numbers of data points per epoch, trial number, and run, respectively, constituting the time domain dataset. Signal is further filtered using band pass filtered in the range of 0.5–30 Hz. For every task, that is, left imagery, right imagery, and state of rest for C3, C4, and Cz, two sets of data are prepared one having spectral content in range of 7–14 Hz and the other in the range of 17–26 Hz. Buttorworth filter with order six is selected to filter the signal vector of length 625 points at a time. All eighteen sets of three-dimensional data mentioned above are filtered producing thirty-six sets of filtered data of appropriate size. Filtered signal is feed to feature extractor which invokes eleven features extracting techniques for two different bands of signal thus resulting in a total of twenty-two features. Features are extracted for *μ* and ß bands of C3 for left imagery tasks, C3 for right imagery tasks, C4 for left tasks, and C4 for right imagery tasks. These features are computed using different algorithms of time frequency representations. Details of these techniques are mentioned in [15, 28].

**Figure 2:**
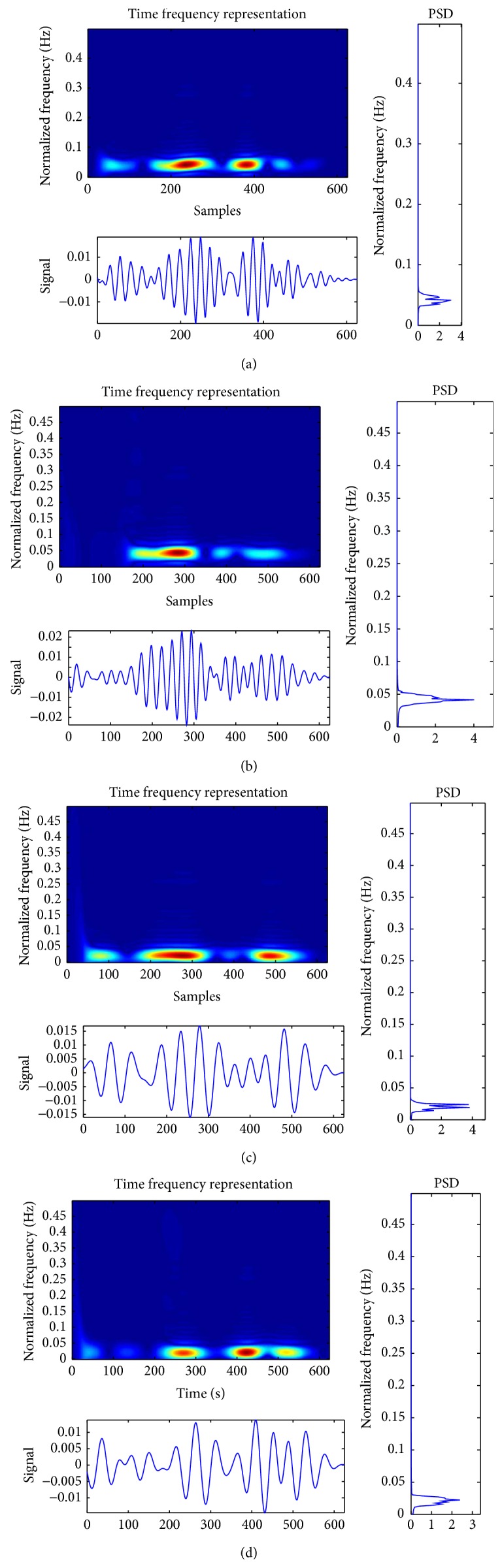
(a) Born Jordan feature for ß band in C3 channel for arbitrary trial corresponding to right task. (b) Born Jordan feature for ß band in C4 channel for arbitrary trial corresponding to right task. (c) Born Jordan feature for *µ* band in C4 channel for arbitrary trial corresponding to left task. (d) Born Jordan feature for *µ* band in C4 channel for arbitrary trial corresponding to right task.

The TFR features used in classification of MI based tasks in this experiment are listed in Table 1. After computing different features in order to form feature vector, absolute value of features is computed. For extracting features, a data vector of length 1.25 second is taken with sampling frequency of 500 Hz. This is also chosen as epoch length and for every processing, a signal vector of 625 points is used at a time. We have used data corresponding to C3 and C4 channels for task discrimination as used in [29].  Figure 2 shows Born Jordan feature computed for arbitrary trials. These features have been computed using time frequency toolbox of MATLAB [24]. Maximum value of the absolute value of feature value (TFRs) obtained for every epoch of data for various features is determined. Then mean value is computed for every trial of each type of tasks. Further concept of ERS and ERD is used in forming feature vector as mentioned in [15, 26] which states that there is ERS of the *μ* rhythm on the contralateral side and a slight ERS in the central ß rhythm on the ipsilateral hemisphere. This hemispheric asymmetry reflected in the EEGs is exploited to differentiate the task desirably. To exploit the concept of hemispheric asymmetry with regard to *μ* and ß bands, we combined feature matrix obtained with respect to *μ* and ß bands to form a pattern, which is likely to inherit the property of ERS and ERD as mentioned in [15]. This resulted in a feature matrix of dimension 160 × 22 where there are twenty-two features using two bands of signal with eleven different features for eighty each of the two classes for every session. In our present work we used two sessions at a time for purpose of classifications. Thus a composite feature matrix of dimension 320 × 22 is obtained. Different techniques of TFR are extensively used in the area of BCI [15, 30–39].

### 2.3. Clustering

Clustering of a data population involves grouping of abstract unlabelled patterns into groups called clusters such that patterns in same cluster are similar to each other and dissimilar to patterns from other clusters. An unlabelled set of sample patterns comprising a population is given as input to a clustering algorithm, which partitions the input patterns into groups called clusters and labels the samples accordingly [13].

The most widely used clustering algorithm, that is, *K*-means algorithm, takes *K* as input and partitions dataset of *N* objects into* K clusters*, where *K* < *N*. Cluster similarity is measured with respect to the dissimilarity between a data point and mean value of the patterns (centroid) in cluster. In general, square-error criterion is used as criterion function, given as

(1) $$\begin{array}{c} D=\overset{K}{\underset{j=1}{\sum }}\;\underset{p\in C_{j}}{\sum }{\left|m_{j}-p\right|}^{2}, \end{array}$$

where *D* is sum of square-error for all patterns in dataset, *m*_*j*_ is mean of pattern in *j*th cluster *C*_*j*_, and *p* represents pattern or point in cluster *C*_*j*_.

Starting with a set of randomly selected cluster centres, the centres are iteratively updated with aim of minimizing criterion function, *D*. In other words *K*-means can also be viewed as optimization strategy, which searches for appropriate cluster centre so as to minimize criterion function, *D* given by (1), based on initial cluster centres given as input to the *K*-means. Conventional *K*-means is very sensitive to the initial selected centers. Several methods have been reported in literature to solve the cluster centre initialization (CCI) problem. The limitations of conventional *K*-means are as follows.

- Objective function of *K*-means, as given by (1), is convex [40]; hence it may contain local minima. Consequently, there is possibility of convergence to the local minima.
- Using conventional *K*-means algorithm we are not able to determine global solution to the problem; that is, clustering as output of *K*-means is highly dependent on initial cluster centres and hence gives local solutions to clustering problem [40–42].

To avoid premature convergence to the local optimal point, in the present work, *K*-*means* algorithm has been formulated as a global optimization problem in the present work. Approaches for solving the optimization problem can be broadly classified into two groups, that is, gradient based and population based evolutionary approach. Though gradient based methods quickly converge to an optimal solution, it fails for nondifferentiable or discontinuous problems. It is not applicable even if the objective function is not completely known due to limited knowledge, which is very likely for real time applications. In this context, EA based optimization technique has been found to be quite effective for multimodal objective functions. High level of dynamism in EEG signal requires techniques that could address complexities such as dynamism and uncertainty. In this work we have used three EA based approaches, that is, PSO, GA, and hybrid GA-PSO, to select the initial centres. Both PSO and GA have their own advantages and limitations. PSO and GA are initialized with a group of a randomly generated population and have fitness values to evaluate the population. They update the population and search for the optimum solutions. In contrary to GA, PSO does not have genetic operators like crossover and mutation. In PSO, particles update themselves with their internal velocity and they also have memory (all particles remember their previous best position). The information sharing mechanism in PSO and GA is also significantly different [43]. In GAs, chromosomes share information with each other. So the whole population moves towards an optimal area as a group. In PSO, only global best particle gives out the information to others and hence it follows one way information sharing mechanism. In PSO all the particles tend to converge to the best solution faster in comparison with GA. Angeline compared between GAs and PSOs and has suggested in [44] that a hybrid of the standard GA and PSO models could furnish better result. Motivated by this, a hybrid GA-PSO based clustering algorithm has been proposed, which combines standard position and update rules of PSO with operators of genetic algorithm: selection, crossover, and mutation as mentioned in [45]. The tuning parameters of both the optimization and the clustering technique are selected based on a series of pilot runs to determine the best possible values for higher classification accuracy.

### 2.4. Swarm Representation

In order to use PSO to solve clustering problem each individual particle position represents *K* cluster centres consisting of *N*∗*K* real numbers in a *K* × *N* matrix, where first row represents first cluster centre and second represents second cluster centre, and so on.

### 2.5. Swarm Initialization

In this step each particle swarm encoded with cluster centre is initialized by randomly choosing *K* data points. Each particle is represented by *K* × *N* matrix. This process is repeated *P* times, where *P* is number of particles.

### 2.6. Fitness Computation

Each particle in consideration is given as input to *K*-means and each point *p*_*i*_ in the data set is assigned to its nearest cluster *C*_*j*_ with *m*_*i*_ as centre. Then a new cluster mean is obtained to get new cluster centre which then replaces the particle in consideration.

Using the above newly obtained cluster centre fitness of the particle is calculated using (1).

### 2.7. Update *P*_best_ and *G*_best_

In this step, each particle updates its personal best position denoted by *P*_best._ and the associated fitness value. *P*_best_ contains each individual particle's best position seen up to the current generation. It is updated as follows.

- For each particle
  - compare particle's current fitness value with fitness value of *P*_best_;
  - if current fitness value is better than fitness value of *P*_best_, then set *P*_best_ value equal to the current position of particle and the update the fitness value associated with it equal to particle current fitness value;
  - otherwise, do nothing.

Updation *G*_best_ involves positioning of particle with best position seen up to current generation. In the first generation of PSO, *G*_best_ and associated fitness are equal to the position and fitness of particle with best fitness value. In the subsequent generations, *G*_best_ is updated as follows.

- For each particle in swarm
  - compare the fitness value of *P*_best_ with the fitness value *G*_best_;
  - if fitness value of *P*_best_ is better than the fitness of *G*_best_, then set *G*_best_ to *P*_best_ and fitness value of *G*_best_ is equal to fitness value of *P*_best_;
  - otherwise, do nothing.

### 2.8. Position and Velocity Update

Half of the worst performing particles (let them be called *P*_2_) are eliminated on basis of their fitness. Remaining half of best performing particles (let them be called *P*_1_) undergo position and velocity update as in standard PSO, given by (2).

(2) $$\begin{array}{c} v\left(t+1\right)=w\times v\left(t\right)+c_{1}\times \left(p\left(t\right)-x\left(t\right)\right)+c_{2}\times \left(g\left(t\right)-x\left(t\right)\right)\\ x\left(t+1\right)=x\left(t\right)+v\left(t+1\right), \end{array}$$

where *x*(*t*), *x*(*t* + 1) is the position of the particle at iteration *t* and *t* + 1, respectively. *p*(*t*) is the personal best (*P*_best_) position of the particle *x*(*t*) found so far. *g*(*t*) is the global best (*G*_best_) position of the any of the particle found so far. *v*(*t*) is the velocity of the particle *x*(*t*) to update its position. *w*(*t*) represents confidence in its own movement or inertial weight. *c*_1_ is a constant called cognitive parameter. *c*_2_ is a constant called social parameter. *w*(*t*) varies with iterations according to equation given below:

(3) $$\begin{array}{c} w\left(t\right)=\frac{\max\_\text{iterartions}-t}{\max\_\text{iterartions}}. \end{array}$$

### 2.9. Selection

Remaining half, that is, *P*/2 particles, are generated from particles that have undergone position and velocity updating using appropriate selection techniques. In this work we have used tournament selection strategy for reproduction or forming the mating pool. In tournament selection “*m*” individuals are randomly selected from the population, where “*m*” is called tournament size. The individual with best fitness among “*m*” individuals is selected into mating pool. Advantage of tournament selection over roulette wheel selection is that it does not depend on negative fitness values and whether a problem is a maximization or minimization problem unlike roulette wheel selection. Also it is not fully biased towards fittest individual in population as in the case of Roulette wheel selection.

### 2.10. Crossover and Mutation

The particles generated in above step using tournament selection go through velocity propelled averaged crossover (VPAC) [45] and mutation. In VPAC, two child particles are produced such that their position is between their parents but is accelerated away from their current direction (by adding negative velocity). VPAC crossover is able to create necessary diversity in particles to make searching process more effective. New children are obtained based on

(4) c1x=p1x+p2(x)2−θ1×p1v,c2x=p1x+p2(x)2−θ2×p2v,

where *c*_1_(*x*) and *c*_2_(*x*) are positions of child 1 and child 2, respectively, *p*_1_(*x*) and *p*_1_(*x*) are positions of parent 1 and parent 2, respectively, *v*_1_(*x*) and *v*_2_(*x*) are velocities of parent 1 and parent 2, respectively, *θ*_1_ and *θ*_2_ are uniformly distributed random numbers between 0 and 1, and child particle retains their parents velocity; that is, *c*_1_(*v*) = *p*_1_(*v*) and *c*_2_(*v*) = *p*_2_(*v*).

Then particles, after going through velocity propelled averaged crossover, undergo mutation as described in [46]. In this step each chromosome undergoes mutation with fixed small probability, *μ*_*m*_. For mutating a chromosome, whose clustering metric is *M*, a number ∂ in the range (−*R* to +*R*) is generated with uniform distribution, where *R* is calculated as follows:

(5) $$\begin{array}{c} R=\left\{\begin{array}{cc} \frac{M-M_{\max}}{M_{\max}-M_{\min}}, & \text{if}\;M_{\max}>M,\\ 1, & \text{if}\;M_{\max}=M_{\min}, \end{array}\right. \end{array}$$

where *M*_min⁡_ and *M*_max⁡_ are the minimum and maximum values of the clustering metric, respectively, in the current population. If the minimum and maximum values of the data set along the *i*th dimension (*i* = 1,2,…, *n*) are *x*_min⁡_^*i*^ and *x*_max⁡_^*i*^, respectively, and the position to be mutated is *i*th dimension of a cluster with value, *x* ^*i*^, then after mutation value becomes

(6) $$\begin{array}{c} x^{i}+\partial \times \left(x_{\max}^{i}-x^{i}\right)\;\text{if}\;\partial >0,\\ x^{i}+\partial \times \left(x^{i}-x_{\min}^{i}\right)\;\text{otherwise}. \end{array}$$

This scheme of mutation provides perturbation in a maximum range to strings either when they have the largest value of *M* in the population (i.e., *M* = *M*_max⁡_) or when all the strings have the same value of the clustering metric (i.e., *M* = *M*_min⁡_ − *M*_max⁡_). On the other hand, the best string(s) in the population (i.e., the one with *M* = *M*_min⁡_) is not perturbed at all in the current generation. Moreover, the perturbation is such that the mutated centres still lie within the bounds of the data points.

### 2.11. Termination Criteria

The process of fitness computation, updating of personal best, global best, velocity and position, and crossover and mutation repeated for maximum number of iterations. Position of global best particle at the last iteration gives solution to the clustering problem.

## 3. Results

The appropriateness of the proposed hybrid evolutionary technique in classifying two class MI tasks has been evaluated in this section. The objective functions of GA based *K*-means clustering algorithm, PSO based *K*-means clustering, and GA-PSO based *K*-means clustering algorithm were modified to give misclassification as output for the optimization problem. The formulation of the objective function in terms of classification accuracy allows using the optimization based *K*-means clustering for maximizing classification accuracy. The class information is not used for clustering but for obtaining the solution of the optimization problem. The class information is further used to test the performance of the clustering result. The data sets for each subject were separately evaluated using the evolutionary algorithms and conventional *K*-means clustering. Keeping population size as 1000, algorithms were executed for 100 iterations. For GA based *K*-means classifier algorithm crossover probability, *μ*_*c*_ = 0.65, and mutation probability, *μ*_*m*_ = 0.08, were taken. For PSO based *K*-means clustering algorithm cognitive parameter, *c*_1_ = 1.49, and social parameter, *c*_2_ =1.49, and inertial weight according to (3) were taken. For GA-PSO based *K*-means classifier algorithm parameters crossover probability, *μ*_*c*_ = 0.65, mutation probability, *μ*_*m*_ = 0.08, cognitive parameter, *c*_1_ = 1.49, social parameter, *c*_2_ = 1.49, and inertial weight according to (3) were taken. Above parameters are selected by hit and trial method.

**Figure 3:**
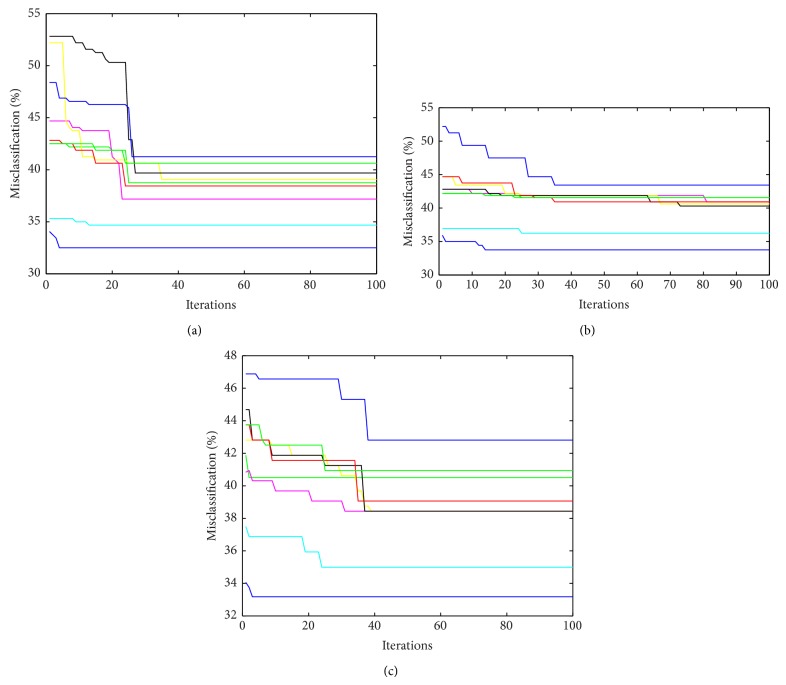
(a) Variation in misclassification (%) with iterations for GA-PSO based *K*-means clustering for different subjects. (b) Variation in misclassification (%) with iterations for GA based *K*-means clustering for different subjects. (c) Variation in misclassification (%) with iterations for PSO based *K*-means clustering for different subjects.

Table 2 shows the results for performance of GA based *K*-means classifier algorithm, PSO based *K*-means classifier and GA-PSO based *K*-means classifier algorithm based on accuracy obtained on test set for each of nine subjects.  Figure 3 shows the variation of misclassification (%) with number of iterations for an arbitrary subject. It can be seen that the hybrid technique not only achieves a higher classification but also achieves the same with lesser iterations. The lesser execution time of the hybrid technique makes it suitable for real time BCI application, where imagery signal needs to be classified at a very fast rate. Further we used statistical test on the results to test the significance of the result.  Table 3 indicates the average ranking of clustering algorithms based on the Friedman's test and Table 4 shows various statistical values from Friedman and Iman-Davenport tests indicating rejection of null hypothesis. The rejection of null hypothesis indicates similarity in the superiority of the proposed hybrid GA-PSO method over other techniques, across all the subjects.

## 4. Conclusion and Discussions

It can be observed from Table 2 that except for subject 5 and subject 6 GA-PSO based *K*-means classifier algorithm is able to outperform or is comparable in terms of average accuracy values obtained for 100 executions of each algorithm. The performance of proposed clustering algorithm may further increase if it is executed for more number of iterations and with greater size population. The hybrid GA-PSO algorithm has been used to search cluster centres in the search space such that criterion function, *D*, given by (1), is minimized. The knowledge that the mean of points belonging to same cluster represents the cluster centre has been used for improving the search capability of optimization based clustering method. Floating point representation has been adopted to represent candidate solutions (in GA candidate solutions are known as chromosomes and in PSO they are known as particles), since it is found to be more appropriate and natural for encoding the cluster centres. It can be observed from results that GA-PSO based *K*-means clustering algorithm performs better or at least gives comparable performance with GA based *K*-means clustering algorithm and PSO based *K*-means clustering algorithm.  Figure 3 displays the convergence characteristics of the three optimization based clustering algorithms for all the three cases. It is clear that the hybrid technique (Figure 3(a)) is able to converge to the final solution quite early (lesser iterations and computational time) as compared to the isolated GA (Figure 3(b)) and PSO (Figure 3(c)) based techniques. Thus the hybrid technique not only achieves a higher classification but also achieves the same with lesser iterations. For validation of the GA-PSO based *K*-means clustering, statistical analysis is also performed along with the experimental analysis. A statistical analysis is performed to demonstrate the significant differences among the results obtained using the different algorithms. To prove the same, Friedman and Iman-Davenport statistical tests are carried out. The nonparametric statistical test allows checking the significance and repeatability of the results. In other words, Table 2 indicates the average ranking of clustering algorithms based on Friedman's test using sum of intracluster distance (average) parameter and the critical value obtained for Friedman test *X* (0.05, 3) is 7.814733. The *P* values computed by the Friedman test and the Iman-Davenport test are given in Table 4, both of these tests reject the null hypothesis and support the presence of significant differences among the performance of all clustering algorithms used in this work.

Basically MI based BCIs are composed of two stages: feature extraction and feature classification [47, 48]. The ability to analyze EEG is limited by methods that require stationary epochs of data. Stationary time-series techniques such as Fourier transform for feature extraction cannot be used for EEG signal; hence joint time-frequency analysis is required [30]. TFR is based on the principle of extracting energy distribution on time versus frequency. In this way different frequency components are localized at a good temporal resolution [31]. TFRs can provide amplitude and phase spectra in both frequency and time domain [49]. TFRs methods have been proposed for EEG [30–32], electromyogram (EMG) [50], and EGG [51] signal analysis. TFRs offer the ability to analyze relatively long continuous segment of data even when dynamics are rapidly changing. One approach to the accurate analysis of nonstationary signal is to represent the signal as a sum of waveforms with well-defined time frequency properties. Time frequency techniques are further classified into two classes, namely, linear and bilinear time frequency representations. In our analysis we have used techniques from both classes. Details of these techniques are mentioned in [28]. For classifying MI based task a hybrid GA-PSO based *K*-means clustering algorithm has been used in this work along with GA and PSO based *K*-means clustering to compare and hence to check its suitability in classification problems of MI based task. Our result shows that combination of GA and PSO forming hybrid GA-PSO based *K* means clustering algorithm almost outperforms both the GA and PSO based *K*-means clustering algorithm. Enhanced performance achieved in case of hybrid GA-PSO based *K*-means clustering can be explained in terms of the benefit that PSO and GA facilitates. The algorithm is designed such that GA facilitates a global search and PSO facilitates a local search. The hybrid GA-PSO approach merges the standard velocity and position update rules of PSO with that of GA's operations (i.e., selection, crossover, and mutation) [45]. Data is likely to get affected by level of degree of attention of the subject performing the task and the changes in their concentration can affect the result adversely. Therefore few sets of pretraining prior to actual recording and ensuring that subject take sufficient rest before the actual recording becomes essential. In this analysis we have used fixed frequency band (i.e., *μ* and ß bands) for every subject for ERD/ERS discrimination to reduce the complexity of the system. Selecting varying *μ* and ß bands as used in [15] is likely to give better result. Overall performance will also depend on reliability and performance of other techniques used such as data filtering, feature extraction, and normalization.

## Figures and Tables

**Table 1:** Features used for classification with their type.

Type	Feature
Linear time frequency processing	Short time Fourier transform

Bilinear time frequency processing (Cohen's class)	Born Jordan
	Choi-Williams distribution
	Pseudo-Wigner-Ville distribution
	Smoothed pseudo-Wigner-Ville distribution
	Wigner-Ville distribution
	Zhaos-Atlas-Marks distribution

Bilinear time frequency processing (Affine class)	Unitary Bertrand distribution
	D-Flandrin distribution
	Scalogram for Morlet wavelet
	Smoothed pseudo-Affine-Wigner distribution

**Table 2:** Classification performance against different *K*-means based clustering.

Subject	*K*-means	GA based *K*-means	PSO based *K*-means	GA-PSO based *K*-means
Subject 1				
Average (%)	57.19	58.32	59.48	60.42
Standard deviation	3.271	0.229	1.484	0.137
Subject 2				
Average (%)	58.75	66.08	66.81	67.46
Standard deviation	2.734	0.435	0.218	0.105
Subject 3				
Average (%)	62.50	63.89	64.60	65.25
Standard deviation	4.872	0.295	0.177	0.746
Subject 4				
Average (%)	50.62	58.18	61.18	61.38
Standard deviation	3.979	0.518	2.771	0.660
Subject 5				
Average (%)	56.87	58.18	60.33	59.60
Standard deviation	2.794	1.107	2.989	1.714
Subject 6				
Average (%)	57.19	58.50	60.28	57.37
Std. deviation	5.552	0.868	5.148	1.782
Subject 7				
Average (%)	53.44	58.29	59.96	60.82
Standard deviation	4.492	1.271	1.427	1.410
Subject 8				
Average (%)	54.06	58.32	58.62	59.00
Standard deviation	2.686	0.220	0.705	0.826
Subject 9				
Average (%)	50.62	55.01	55.88	57.40
Standard deviation	2.794	2.561	2.371	1.736

**Table 3:** Average ranking of clustering algorithms based on Friedman's test with a critical value: *X*(0.05,3) = 7.814733.

Algorithms	*K*-means	GA based *K*-means	PSO based *K*-means	Hybrid GA-PSO based *K*-means
Ranking	4	2.89	1.78	1.33

**Table 4:** Friedman and Iman-Davenport statistical tests.

Method	Statistical value	*P* value	Hypothesis
Friedman	23.13333	3.79 × 10^−5^	Rejected
Iman-Davenport	47.8621	<0.00001	Rejected
